# A prevertebral desmoplastic fibroblastoma presenting with sleep‐related breathing dysfunction

**DOI:** 10.1002/ccr3.3897

**Published:** 2021-02-10

**Authors:** Cezary Bielak, Jake Ahmed, Tehreem Atif, Uma Nair, Arvind Arya, Hisham Zeitoun, Ligy Thomas

**Affiliations:** ^1^ Ysbyty Glan Clwyd Betsi Cadwaladr University Health Board Wales UK

**Keywords:** desmoplastic, fibroblastoma, prevertebral, retropharyngeal

## Abstract

Desmoplastic fibromas exceedingly rarely present in the retro/parapharyngeal space but should be considered in differential diagnosis for benign lumps in these anatomical regions.

## Graphical abstract

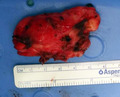

## 1 INTRODUCTION

Parapharyngeal and retropharyngeal masses are known to be associated with dysphagia and sleep‐related breathing disorders ^1^—yet benign pathology presenting in this anatomical subsite remains rare with lipomas appearing to be the most commonly encountered lesion.^2^ Additionally, the retropharyngeal space is classically difficult to access for diagnostic sampling (needle aspirate or core biopsies) due to its depth and presence of adjacent critical neurovascular structures, thus histological diagnosis is typically only discovered postoperatively.^3^ We present a case of retropharyngeal desmoplastic fibroblastoma (DF) presenting with new onset of snoring and dysphagia.

## 2 CASE REPORT

A 30‐year‐old man was referred by his general practitioner to our department for review of recurrent tonsillitis. While awaiting a tonsillectomy, the patient developed new‐onset snoring and sense of airway obstruction which relieves on neck extension and a lump in his throat. He had no significant past medical history and denied smoking or drinking alcohol.

Clinical examination including flexible nasendoscopy demonstrated a firm, smooth swelling arising from the left lateral pharyngeal wall extending superiorly to the level of the soft palate and inferiorly to the level of epiglottis. The oral cavity, oropharynx, larynx, and both piriform fossae appeared normal.

**FIGURE 1:**
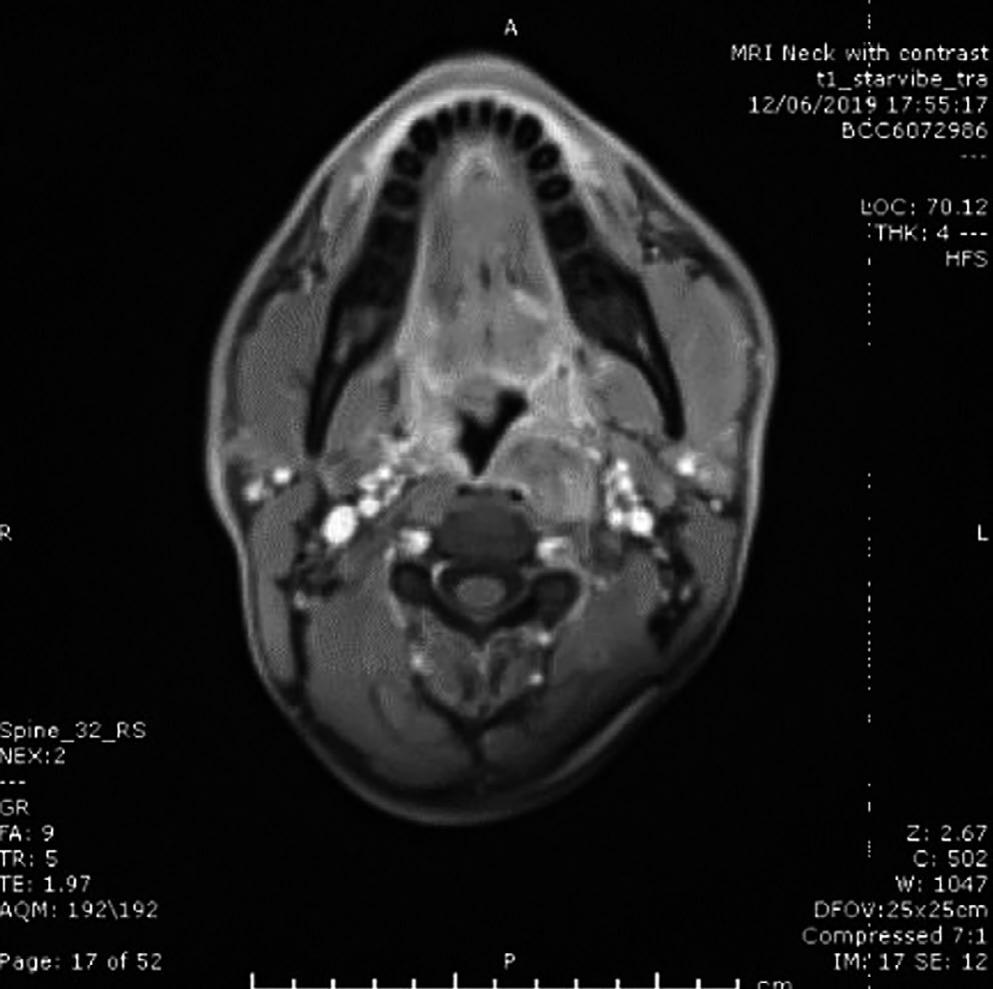
Sagittal T1 MRI section showing retropharyngeal soft tissue lesion (white arrow)

**FIGURE 2:**
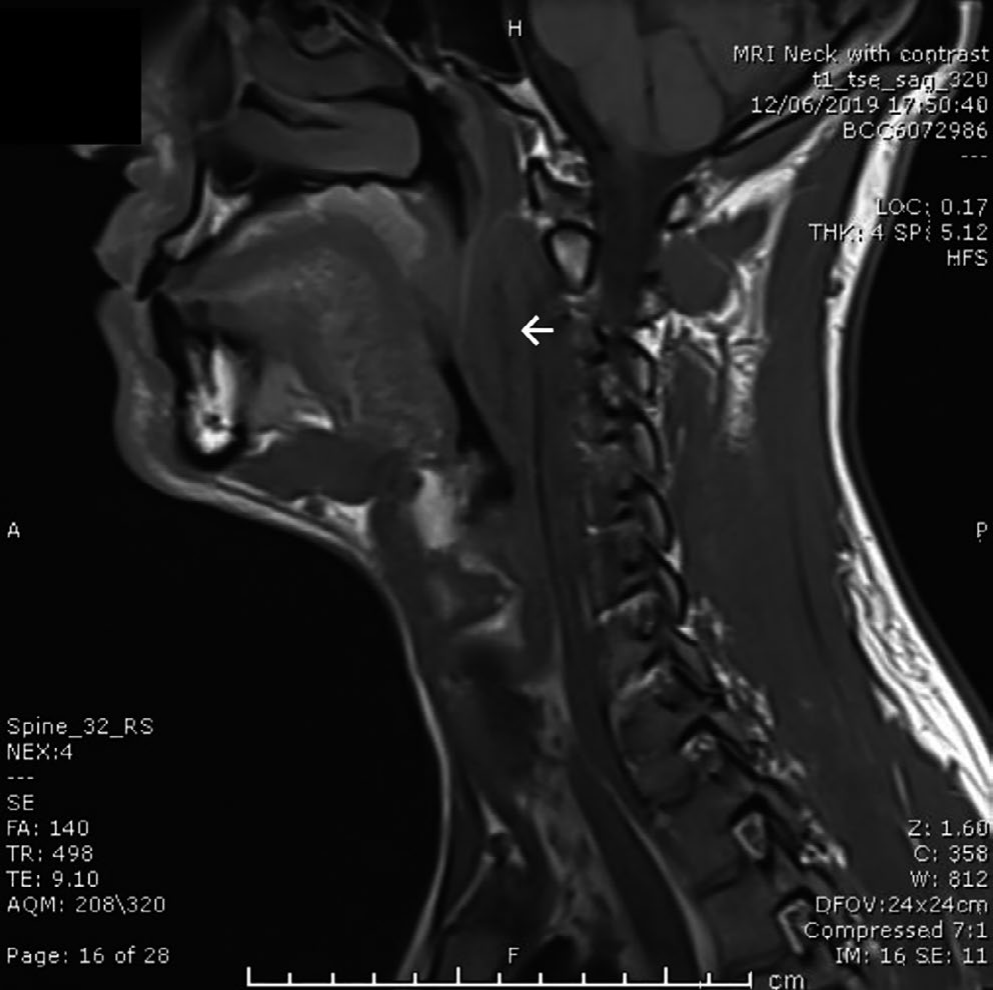
Coronal MRI section showing T1 image contrast‐enhanced

A magnetic resonance imaging scan (Figures 1 and 2) showed *a well‐defined 2.9 x 1.9 cm low signal mass inseparable from the left capitis longus muscle on T1 precontrast images with anterior displacement of the well‐preserved prevertebral fat and no locally aggressive features. There was uniform enhancement with contrast and no diffusion restriction. Overall, the appearances were in keeping with a benign soft tissue tumor*.

Contrast CT reported an absence of calcification or enhancement supporting a benign process with no aggressive features. The patient underwent examination under anesthetic and biopsy of the pharyngeal mass and the fine needle aspiration of the neck lump reported only inflammatory cells and numerous benign squamous epithelial cells with occasional macrophages.

The case was discussed at the local head and neck multidisciplinary team meeting with a resulting recommendation of local excision of the primary lesion with the aim of relieving clinical symptoms. The patient was appropriately consented and listed for an external approach neck exploration with excision of lesion.

**FIGURE 3:**
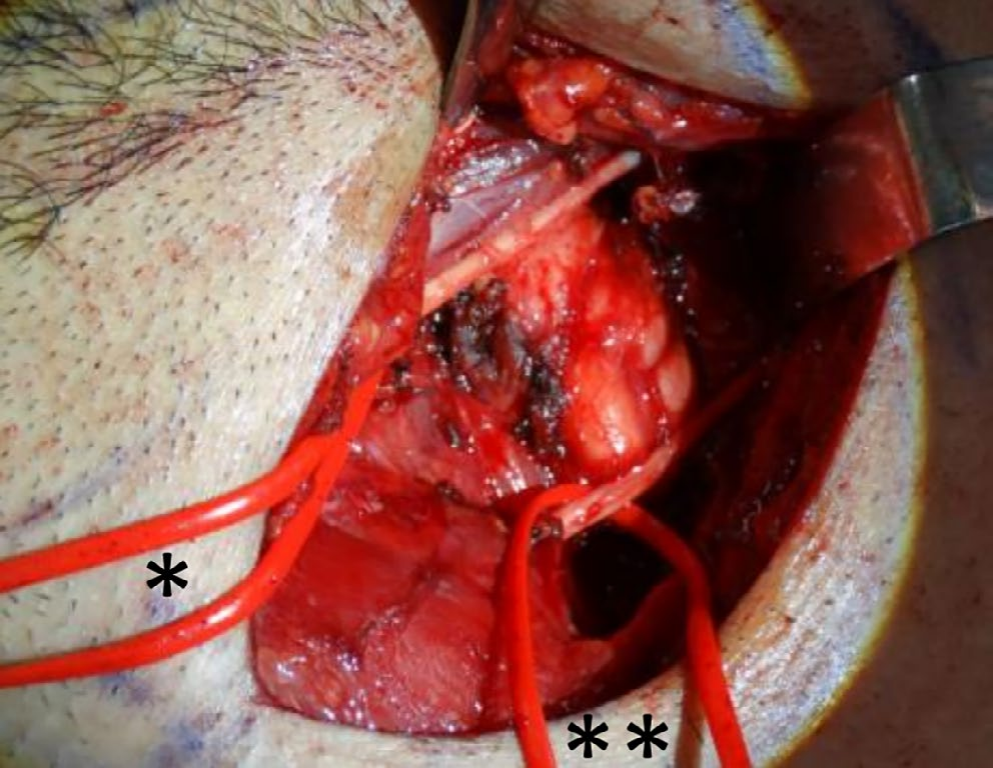
Showing lesion in situ in the retropharyngeal space (*loop around left hypoglossal nerve, **loop around left superior laryngeal artery)

**FIGURE 4:**
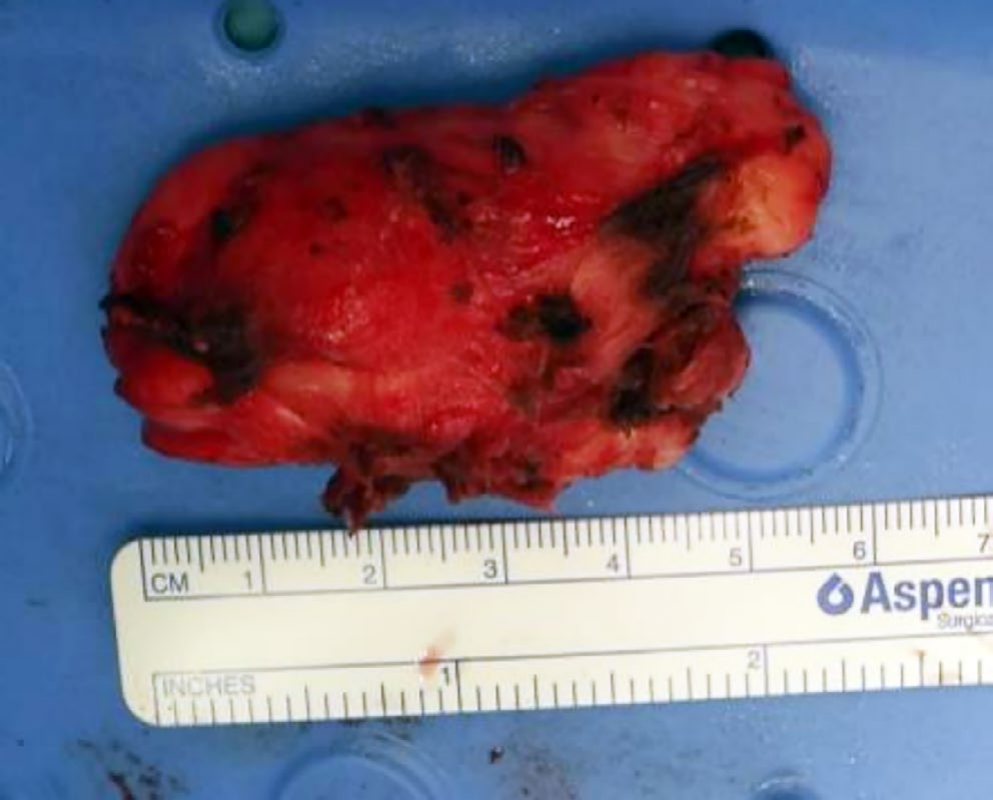
Lesion excised en block measuring 59mm in longest dimension

The lesion was accessed by horizontal skin crease incision, clearing level 2a soft tissue, identifying neurovascular structures (great vessels which were retracted laterally, dissecting and protecting hypoglossal, accessory and superior laryngeal nerves). Mass was dissected superiorly and freed from the prevertebral muscles. Intraoperative specimens (Figures 3 and 4) consisted of the primary lesion (6cm x 3.5cm x 2cm) originating from the left prevertebral muscles, superiorly adjacent to the skull base and inferiorly extending to just below the left common carotid artery bifurcation. Level 2a nodal clearance was done to approach the lesion safely and to ensure safety of important neurovascular structures. The mass was removed en bloc preserving all adjacent neurovascular structures.

**FIGURE 5:**
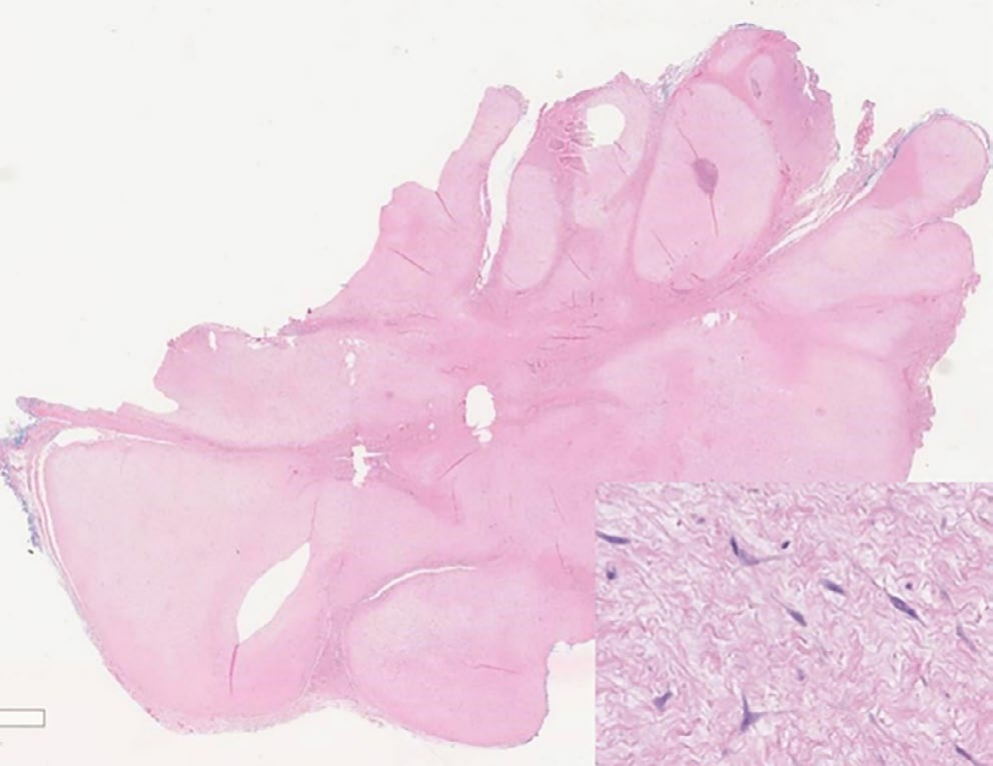
Histological slides showing sparsely cellular lesion with stellate cells. (Scanning magnification with inset 10× magnification)

Histological analysis (Figure 5) of the excised mass reported: *a circumscribed multilobulated tumor composed of sparsely cellular fibromyxoid nodules with scattered bland spindle or stellate cells. No significant nuclear pleomorphism or mitotic activity was seen, and no necrosis was identified. At its periphery the tumor was found to have infiltrated skeletal muscle tissue. The specimen was focally positive for SMA and h‐caldesmon, but negative for MUC4, desmin, EMA, S100 protein, SOX10, ERG, AE1/AE3, and beta‐catenin. Suggestive of a desmoplastic fibroblastoma, although with an exceptional location and multinodular architecture*.

The patient's postoperative period was uneventful except for mild hypoglossal neuropraxic symptoms in the immediate post op period which recovered completely within 2 months.

## 3 DISCUSSION

Desmoplastic fibroblastomas (DFs) are uncommon benign fibrous soft tissue tumors typically occurring between the ages of 40 and 69 and are rarely observed in the tongue, palate, and neck.^4^ DFs are composed of fibroblasts/myofibroblasts with variable collagen formation and are also known as collagenous fibromas^5^, ^6^ due to their tendency to show abundant collagen formation which has historically been used as a diagnostic clue. Available literature describes a few rare cases of DFs of the hard palate and oral cavity^6^, ^7^ and one case in the hepatic parenchyma. While they are most commonly located on the skin of the upper arms, extremities, head and neck, shoulder, and thigh,^8^, ^9^ there have been no case reports of DFs in the retropharyngeal /parapharyngeal space.

Radiologically, the appearance of DF is variable and although lesions are usually described as heterogenous and well circumscribed,^10^ the degree of variability prevents reliable radiological diagnosis. Definitive diagnosis depends on histopathological analysis. In this case, the gross features of the lesion included a well‐demarcated soft tissue growth with gray‐white to tan cut surface having a firm consistency.^10^ Microscopically a classic DF is composed of stellate to spindle shaped cells having a fibroblastic to myofibroblastic appearance with abundant collagen formation. These cells have moderate amounts of cytoplasm with usually vesicular nuclei and a prominent small nucleolus. The background stroma is fibrous to fibromyxoid with small scattered vessels. Rarely extension into the underlying muscle (27%)^10^ and fat have been reported.^11^, ^12^

This case is unique in its clinical presentation with respect to its location and notable multilobular morphology. Although a major percentage of these tumors are delineated, this case showed extension in the adjacent muscle. Follow‐up studies reveal no recurrences of reported cases of this entity and conservative excision is considered curative.^10^

Commonest non‐inflammatory prevertebral masses are malignant nodes or invading mucosal head and neck malignancies. Other benign lesions in this region can be vascular malformations, neurogenic, lipogenic, and salivary tumors which can extend into the retropharyngeal space from the parapharyngeal space.

## 4 CONCLUSION

Desmoplastic fibromas exceedingly rarely present in the retro/parapharyngeal space and should be considered in differential diagnosis for benign lumps in these anatomical regions. We have not seen pathology of this type originating from the retro/parapharyngeal space reported in the literature but have shown that diagnostic surgical resection can be performed with the corresponding intent to alleviate symptoms. Once excised and histology is clarified, it is reasonable to manage in the outpatient setting with tapered follow‐up for surgical sequelae and eventual discharge.

## 5 ETHICS STATEMENT

Patient consent was obtained for all data in this publication.

## CONFLICT OF INTEREST

None declared.

## AUTHOR CONTRIBUTIONS

Cezary Bielak: wrote abstract, case report, Key clinical message, and manuscript submission.

Jake Ahmed: wrote introduction, case report, discussion, and conclusion and created figures.

Tehrim Atif: wrote case report, histopathology findings and created figures.

Uma Nair: served as lead histopathology consultant, histopathology findings, and manuscript review.

Arvind Arya: served as lead ENT consultant, manuscript review, and editing.

Hisham Zeitoun: served as lead ENT consultant, manuscript review, and editing.

Ligy Thomas: served as team lead consultant, manuscript review, and editing.

## Data Availability

Data sharing is not applicable to this article as no new data were created or analyzed in this study.
